# Enhanced efficacy with reduced toxicity of chemotherapy drug 5-fluorouracil by synergistic treatment with Abnormal Savda Munziq from Uyghur medicine

**DOI:** 10.1186/s12906-017-1685-4

**Published:** 2017-04-07

**Authors:** Tao Yang, Mutalifu Aimaiti, Deqi Su, Weiwei Miao, Bin Zhou, Dilinuer Maimaitiyiming, Abdiryim Yusup, Halmurat Upur, Ainiwaer Aikemu

**Affiliations:** 1 Department of pharmaceutical analysis, Xinjiang Medical University, Urumqi, 830011 China; 2 Central Laboratory of Xinjiang Medical University, Urumqi, 830011 China; 3 Toxicology Department School of Public Health, Xinjiang Medical University, Urumqi, 830011 China; 4 Heart Center, The First Affiliated Hospital of Xinjiang Medical University, Urumqi, 830011 China; 5 Uyghur Medical College, Xinjiang Medical University, Urumqi, 830011 China

**Keywords:** Abnormal Savda Munziq, Cervical carcinoma, Reducing toxicity, Enhancing efficacy

## Abstract

**Background:**

Abnormal Savda Munziq (ASMq) is a traditional prescription in Uyghur Medicine, and its treatment of complex diseases such as tumors and asthma has been proven to be effective in Uyghur medical clinical practice. The efficacy-enhancing and toxicity-reducing properties of ASMq were studied on mice with transplanted cervical cancer (U27) tumors, which were treated with 5-fluorouracil (5-FU) in this work.

**Methods:**

To investigate the synergistic effect of ASMq and 5-FU on U27 cells, inhibitory effects on cell proliferation were determined through a MTT assay. 48 Kunming mice which were randomly divided in to 6 groups: control group, model group, 5-FU group, 5-FU combine with ASMq low-dose group, 5-FU combine with ASMq medium-dose group, and 5-FU combine with ASMq high- dose group, the inhibition rate of the tumor, the viscera indexes, and the content of serum tumor necrosis factor-α (TNF-α), alanine aminotransferase (ALT) and aspartate aminotransferase (AST) were determined. The expression levels of transforming growth factor-β1 (TGF-β1) and human papillomavirus type 16 E2 (HPV16 E2) protein were assessed by Western blot. Pathological changes in the liver were observed.

**Result:**

The inhibition rates of tumors, the 5-FU + ASMq.H group(80.64%), 5-FU + ASMq.M group (90.67%), 5-FU + ASMq.L group (72.03%) and 5-FU group (66.89%), clearly indicated that the effects of tumor inhibition. The thymus index and spleen index were increased, and the serum concentration of TNF-α increased while ALT and AST concentrations were decreased, and TNF-α protein expression were increased while TGF-β1 and HPV16 E2 were decreased. ASMq might can improve livers central vein hyperemia and interstitial edema, and preserve the radial structure of the hepatic cords.

**Conclusions:**

The results suggested that ASMq might reduce toxicity and enhance the efficacy of the chemotherapeutic drug 5-fluorouracil in the treatment of cervical carcinoma.

## Background

Cervical cancer is the fourth most common malignancy cancer among women allover the world. According to the data from 2012, 528,000 cases of cervical cancer were detected throughout the world annually, caused 266,000 deaths per year. 85% of these cervical cancer cases occured in developing countries which is the top cause of death in these countries [1, 2]. As a common method used on comprehensive cancer treatment, chemotherapy has made inspiring achievements. In recent years, a new type of chemotherapy called neoadjuvant chemotherapy has been proposed. However, chemotherapy drugs that killing tumor cells may also have side effects, such as reducing immune function and increased organ toxicity. In addition, chemotherapy also can cause the drug resistance of tumor. Recent studies have shown that chemotherapy treated with traditional Chinese medicine could improve the efficiency of tumor inhibition, while mitigating their side effects [3–5].

Abnormal Savda Munziq [6, 7] is made from 10 kinds of medicinal herbs including *Anchusa ita1ica Retiz, Cordia dichotoma Forst, Ziziphus jujuba Mill, Foeniculum vulgare Mill, Glycyrrhiza uralensis Fisch, Dracocephalum moldavica L, Euphorbia humifusa Willd,* and *Adiantum capillus-veneris L*. Previous studies have shown that ASMq can inhibit the growth of human cervical cancer [8] and other cells, and inhibit the growth of mice transplanted s180 tumor [6, 7]. In addition, Asmq not only has the reversal effect on 5-FU multidrug resistance cell line Bel/Fu cells resistant [9], and low concentrations of AMSq total phenolics(50 μg/mL) has no inhibitory effect on HeLa cells, but also can enhance cisplatin and docetaxel on HeLa cell growth inhibition rate [10].

In this study, a transplanted tumor-bearing mouse model of cervical cancer was proposed and used to investigate the efficacy enhancement and toxicity reduction of chemotherapy drug 5-fluorouracil (5-FU) by Abnormal Savda Munziq. The results of this study would be used for further clinical application of Abnormal Savda Munziq.

## Methods

### Materials

Abnormal Savda Munziq was purchased from Xinjiang Qikanghabo Uighur medicine research co. Ltd. (Urumqi, Xingjiang, China). 5-Fluorouracil was purchased from Tianjin Jinyao Pharmaceutical Industries Co. Ltd. (Tianjin, China). TNF-α was purchased from Boster Biological technology (Wuhan, Hubei, China). AST and ALT were purchased from Nanjing Jiancheng Biological Technology (Nanjing, China). 3-(4, 5-dimethylthiazolyl-2)-2,5-diphenyltetrazolium bromide (MTT) was purchased from Beijing Cellchip Biotechnology Corporation (Beijing, China).

Forty-eight Kunming mice with a body weight of 18-22 g (male and female) were provided by Xinjiang Medical University Laboratory Animal Center (Certificate No. SCXK (Xin) 2011–0004). A mouse strain of cervical carcinoma (U27) was provided by the cell bank of Wuhan University.

### The Abnormal Savda Munziq preparation

The dried *Lavandula angustifolia Mill* and *Foeniculum vulgare Mill* were extracted with 10 times water by steam distillation to get the volatile oil, then collecting the liquid after filtration. After mixing the volatile oil and β-cyclodextrin, the inclusion were dried, grind and filtered. The dried crude powder of *Ziziphus jujuba Mill*, *Anchusa italica Ret*, *Alhagi pseudalhagi (Bieb) Desv* and *Cordia dichotoma G Forst* were mixed and extracted with 10 times water under reflux. After filtration through filter paper, followed by concentration in vacuum and centrifugation, and the supernatant were collected. The dried raw powder of *Glycyrrhiza glabra L*, *Euphorbia humifusa Willd Euphorbia maculata L*, *Melissa officinalis L* and *Adiantum capillus-veneris L* were extracted with 10 times ethanol (65%), and the filtrate were collected after filtration. All the liquid of water extracts were mixed and concentration in vacuum to obtain the extractum with relative density of 1.20–1.25. After dried and grinded, the extractum were mixed with the volatile oil and lactose. With PVP (5%, in 90% ethanol), added the mixture were knead and through a 14 mesh sieve. The granules were obtained after dried and granulated.

### MTT assay for the inhibition of U27 cell growth

The U27 cells growing in logarithmic phase were seeded in 96-well plates at 1.0 × 10^4^ cells per well and allowed to adhere overnight, after which a series of concentrations of ASMq (1.25, 2.5, 5, 10, and 20 mg/mL) and/or 5-FU (3.125, 6.25, 12.5, 25 and 50 mg/L) were added to the well for 24, 48 or 72 h. Controled experiments were carried out by adding ethanol without ASMq to U27 cells. After 24, 48 or 72 h of incubation, 20 μL MTT (5 mg/mL) was added to each well and the cells were re-incubated for another 4 h at 37 °C. After removaling of the supernatant gently, 200 μL of dimethyl sulfoxide (DMSO) was added to each well to solubilize the purple formazan crystals completely. Absorbance values at 570 nm were measured with a microplate reader and were reported as a percentage of growth with respect to the control. Inhibition rate of cell growth was measured using the formula: inhibition rate (%) = [1- OD_570_ (experiment group)/OD_570_ (control group)] × 100. All experiments were repeated in triplicate. The drug concentration that produced 50% inhibition of cell proliferation (IC_50_) was calculated and analyzed for 5-FU, ASMq and the combination.

### Establishment of mouse model bearing U27 cell-induced tumor and grouping

Kunming mice housed in groups of four and given five days to acclimate to the housing facility. Environmental conditions were a temperature of 20 ± 2 °C, humidity of 55 ± 5%, 12-h light and 12-h dark cycle with lights on at 08:00 and off at 20:00. Animals were housed in 290 × 178 × 160 mm cages and given ordinary food and water. At the start of the experiments animals weighed 20 ± 2 g.

Forty-eight Kunming mice were randomly divided into the following groups: 1) control group, 2) model group, 3) 5-fluorouracil (5-FU) alone, 4) 5-fluorouracil combined with a high dose of Abnormal Savda Munziq (5-FU + ASMq.H) group, 5) 5-fluorouracil combined with a medium dose of Abnormal Savda Munziq (5-FU + ASMq.M) group, and 6) 5-fluorouracil combined with a low dose of Abnormal Savda Munziq group (5-FU + ASMq.L) group. There were 8 mice in each group, four male and four female. Except the control group, the U27 cell-induced tumor model was established in all mice in the other 5 groups. Methods: healthy mice were selected from which to aspirate ascites under aseptic conditions. U27 cells was diluted into 1.0 × 10^7^ cfu/mL using normal saline and added to the ascites suspension. Then 0.2 mL of ascites suspension was subcutaneously inoculated into the axilla of the left forelimb of every mouse. The entire process was completed within 30 min. After 24 h, intervention was performed according to the weight of mice: 1) control group: intraperitoneal injection of normal saline (NS) 0.2 mL/10 g once every other day, and intragastric administration of NS 0.2 mL/10 g once every day; 2) model group: intraperitoneal injection of NS 0.2 mL/10 g once every other day, and intragastric administration of NS 0.2 mL/10 g once every day; 3) 5-FU group: intraperitoneal injection of 5-FU 30 mg/kg once every other day, and intragastric administration of NS 0.2 ml/10 g once every day; 4) 5-FU + ASMq.L group, 5) 5-FU + ASMq.M group and 6) 5-FU + ASMq.H group: intraperitoneal injection of NS 0.2 mL/10 g once every other day, and intragastric administration of Abnormal Savda Munziq 2 g/kg, 4 g/kg and 8 g/kg, respectively, once every day for 10 days.

### Expression levels of TNF-α, ALT and AST in mouse serum

After continuous treatment for 10 days, orbital blood was collected and kept at room temperature for 30 min, then centrifuged at 3000 rpm for 20 min before the supernatants were collected. The expression levels of TNF-α, ALT and AST in mouse serum were determined according to the protocols of the corresponding kit.

### Determination of viscera weight, viscera index and inhibition rate of tumor

The length (A) and the shortest(B) diameter of the tumor were measured once every three days, and the tumor volume was calculated according to the formula V = AB [2]/2. The tumor growth curve was according to each group of tumor-bearing nude mice. After continuous treatment for 10 days, the mice were terminated. The thymus, spleen, liver and kidneys were immediately separated. The surrounding connective tissue and fat were excluded. The organs were dried of surface moisture using filter paper, and weighed. Viscera indexes for the thymus, spleen, liver and kidney were calculated by the following formula: organ index = organ weight (mg)/body weight (g). The inhibition rate for each group was obtained using the following formula: inhibition rate of tumor = (average tumor weight in model group - average tumor weight in experimental group)/average tumor weight in model group.

### Analysis of HPV16 E2 and TGF-β1 protein expression in tumors by western blot

Denaturation of protein samples and gel electrophoresis.

A 10% separation gel (4 mL) and a 4% stacking gel were prepared for gel electrophoresis. The frozen protein samples were thawed on ice immediately prior to use. The protein content was quantified using and the appropriate volume of protein sample was mixed with 5× sample buffer, denatured at 95 °C for 10 min, and loaded onto the gel. The electrophoresis apparatus was set to constant voltage of 80 V to allow the samples to run from stacking gel to separating gel at a rate of about 8 V/cm. Then the voltage was increased to 120 V.

Gel transfer membrane and detection.

The polyvinylidene fluoride(PVDF) membrane was immersed in 100% methanol for 2-3 min, The water cleaned membrane rinsed with transfer buffer twice for 2 min and then soaked in transfer buffer. Six layers of filter paper were cut to the same dimensions as the gel and soaked in transfer buffer. The gel containing the samples was washed once with transfer buffer, then the transfer membrane was placed in blocking buffer at room temperature and incubated for 1 h. The primary antibody was diluted with blocking buffer and incubated with the membrane overnight at 4 °C. The reacting membrane was put into a dish and washed three times with Tris Buffered Saline with Tween(TBST) for 10 min, then incubated with secondary antibody by shaking for 1 h at room temperature. The membrane was then washed in TBST to remove the free secondary antibody, exposed, developed and fixed in the darkroom. Relative expression levels were calculated using GAPDH as a reference protein. The experiment was repeated 3 times, and the mean expression was calculated.

### HE staining of liver in mice bearing U27 cell-induced tumors

#### Preparation of liver sections

The liver samples were fixed in 10% formaldehyde and immersed in 70% ethanol for 3 h, 80% ethanol for 2 h, 90% ethanol for 1.5 h, 95% ethanol for 2 h, 100% ethanol for 1 h, successively, then incubated in xylene for 30 min, and embedded in melted paraffin for 3 h until solidification to a wax block at room temperature. Then the paraffin embedded tissue was sectioned with 4 μm thick.

#### Normal HE staining

Paraffin was removed from the sections by soaking them in xylene. They sections were then rehydrated using graded ethanol, then washed with distilled water, and stained with hematoxylin. Hydrochloric acid (1%) containing ethanol was used for differentiation, then samples were rinsed with weakly alkaline water before staining with Eosin. After dehydrated through graded ethanol, the sample were placed in xylene and covered by resin. The morphological changes in the liver cells were then observed using a microscope.

#### Statistical analysis

The results were analyzed by SPSS17.0. Normality test and homogeneity test were performed for all the data. The results were represented by mean ± standard deviation (^−^ *x* ± *s*) and analyzed by a one-way analysis of variance and chi-square test. Analysis of variance was used for comparisons between groups, and *P*<0.05 was considered statistically significant.

The combination of the two drugs and the efficacy of the judgment according to the Chou-Talalay joint index method of the dose effect formula calculated the two drugs combined in a variety of effects of the index. When combination index is 1, the effect between ASMq and 5-FU is considered additive; when combination index is significantly greater than or less than 1, the effect is considered subadditive or supraadditive, respectively. CI = (D1/D_X_1) + (D2/D_X_2), where D1 and D2 are the inhibition rate of ASMq and 5-FU alone on U27 cell growth, Dx1 and Dx2 are the inhibition rate of ASMq and 5-FU in combination on U27 cell growth.

## Results

### Effects of ASMq and 5-FU on the proliferation of U27 cells

**Fig. 1:**
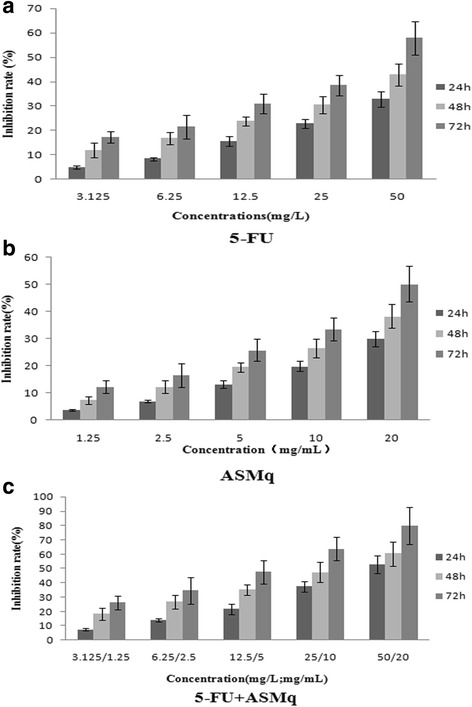
Inhibitory effect of ASMq or/and 5-fluorouracil on the growth of U27 cells. **a** Growth inhibition rate of U27 cells following 24, 48 or 72 h treatment with increasing concentrations of 5-fluorouracil. **b** Growth inhibition rate of U27 cells following 24, 48 or 72 h treatment with increasing concentrations of Abnormal Savda Munziq (ASMq). **c** Growth inhibition rate of U27 cells following 24, 48 or 72 h treatment with increasing concentrations of 5-fluorouracil combined with Abnormal Savda Munziq (ASMq)

To investigate the synergistic effect of ASMq and 5-FU on U27 cells, inhibitory effects on cell proliferation were determined through a MTT assay. First, growth inhibition was measured in U27 cells treated with each of the two compounds individually. The results demonstrated that ASMq and 5-FU could markedly inhibit the proliferation of U27 cells in a significant time- and dose-dependent manner (Fig. 1). In these tests, the ASMq or 5-FU administered for various durations of time had IC_50_ values of 52.45 mg/mL (ASMq: 24 h), 36.78 mg/mL (ASMq: 48 h), 21.12 mg/mL (ASMq: 72 h), 110.12 mg/L (5-FU: 24 h), 81.42 mg/L (5-FU: 48 h) and 35.42 mg/L (5-FU: 72 h). When the two compounds were used in combination, the IC_50_ values for ASMq fell to 18.13 mg/mL, 12.34 mg/mL, and 6.24 mg/mL for incubations of 24, 48 and 72 h, respectively. As the data in Fig. 1 reflect, ASMq can notably strengthen the inhibitory effect of 5-FU on the growth of U27. The IC_50_ values for 5-FU in the presence of ASMq sharply dropped, reaching 46.03 mg/mL, 26.73 mg/mL and 14.21 mg/mL after 24, 48 and 72 h, respectively. An evaluation of the interaction between ASMq and 5-FU and its effect on U27 cell growth (Table 1) yielded a combination index (CI) <1 for different time points (24 h, 48 h and 72 h), suggesting a synergistic effect between the two drugs.

**Table 1:** Analysis of the interaction between the inhibition effect of ASMq in combination with 5-fluorouracil on cell growth by combination index

Times(h)	Combination index	*P* value
24	0.7131 ± 0.1072	<0.05
48	0.6071 ± 0.1021	<0.05
72	0.5321 ± 0.0971	<0.05

### Mice daily activities

We observed mice behaviour, autonomic activities, ingestion, drinking, hairs, faeces and urine daily. There was no secretion in eyes, ears, nose and mouth.

### The effects of 5-FU combined with Abnormal Savda Munziq on the viscera index and the inhibition rate of tumors in mice bearing U27 cell-induced tumors

Comparing with the control group, the thymus index and spleen index of mice in the model group, the 5-FU group, 5-FU + ASMq.H group and 5-FU + ASMq.L group decreased significantly (*P* < 0.05); the liver index of mice in the 5-FU group and different doses groups of 5-FU + ASMq were increased significantly (*P* < 0.05); the kidney index of mice in the different does groups of 5-FU + ASMq were decreased significantly (*P* < 0.05). Comparing with the model group, the thymus and spleen index of mice in the 5-FU group, 5-FU + ASMq.H group and 5-FU + ASMq.L group were decreased significantly (*P* < 0.05); the thymus index and spleen index of mice in the 5-FU + ASMq.M group increased significantly (*P* < 0.05); the liver index in mice in the 5-FU group, 5-FU + ASMq.H group and 5-FU + ASMq.L group were increased significantly (*P* < 0.05); the kidney index in the 5-FU group was increased significantly (*P* < 0.05); the kidney index in the 5-FU + ASMq.H group was decreased significantly (*P* < 0.05). Comparing with the 5-FU group, the thymus index and spleen index of mice different doses groups of 5-FU + ASMq were increased significantly (*P* < 0.05); the liver index and kidney index of mice in different doses groups of 5-FU + ASMq were decreased significantly (*P* < 0.05). These results were summarized and shown in Table 2.

**Table 2:** The effects of 5-FU combined with Abnormal Savda Munziq on the viscera index in mice bearing U27 cell-induced tumors ($\overline{x}$±*s*)

Groups	Dosage g/kg	N	Thymus index (mg/g)	Spleen index (mg/g)	Liver index (mg/g)	Kidney index (mg/g)
Control	-	8	3.965±0.123	5.864±1.989	467.457±189.841	14.4509±4.473
Model	-	8	3.502±0.163^ac^	5.033±2.624^ac^	492.436±364.409^c^	13.913±3.880^ac^
5-FU	0.03	8	2.469±0.140^ab^	3.212±1.103^ab^	637.620±471.837^ab^	14.384±5.538^b^
5-FU+ASMq.H	8	8	3.174±0.087^abc^	4.256±1.928^abc^	547.370±186.089^abc^	12.637±3.249^abc^
5-FU+ASMq.M 4	4	8	3.862±0.088^bc^	5.699±1.435^bc^	504.413±109.636^ac^	13.981±2.884^ac^
5-FU+ASMq.L	2	8	3.112±0.161^abc^	4.148±1.872^abc^	519.447±244.387^abc^	13.960±5.482^ac^

VS control group,^a^ *P*<0.05;VS model group, ^b^ *P*<0.05。VS 5-FU group, ^c^ *P*<0.05

Table 3 shows that the inhibition rate of tumors in mice in the 5-FU + ASMq.H group(80.64%), 5-FU + ASMq.M group(90.67%), 5-FU + ASMq.L group(72.03%) and 5-FU group(66.89%), which clearly indicated that the effects of tumor inhibition. Comparing with the model group, the tumor weight in mice in the 5-FU group and groups of 5-FU + ASMq at different doses, decreased significantly (*P* < 0.05). Comparing with the 5-FU group, the tumor weight in mice in the 5-FU + ASMq.H group and 5-FU + ASMq.M group were decreased significantly (*P* < 0.05).

**Table 3:** Inhibitory effect of Abnormal Savda Munziq on the growth of transplanted cervical cancer tumors in mice when combined with 5-FU ($\overline{x}$±*s*)

Groups	Dosage/(g/kg)	Start animals/End animals	Weight/(mg)	Tumor control rate/%
Control	-	8/8	0.000±0.000	-
Model	-	8/8	2596.45±293.296^b^	-
5-FU	0.03	8/8	859.74±279.018^a^	66.89
5-FU+ASMq.H	8	8/8	502.57±327.528^ab^	80.64
5-FU+ASMq.M	4	8/8	242.31±154.845^ab^	90.67
5-FU+ASMq.L	2	8/8	726.31±514.482^a^	72.03

VS model group, ^a^ *P*<0.05;VS 5-FU group, ^b^ *P*<0.05

**Fig. 2:**
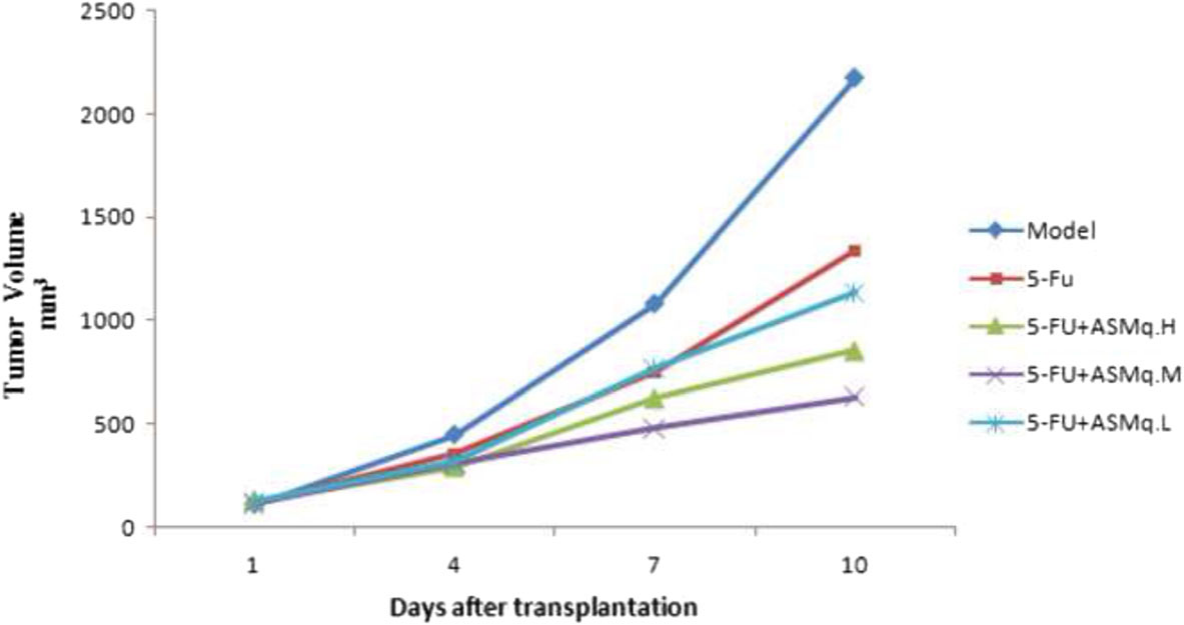
Tumor growth curve

As shown in Fig. 2, after treated with ASMq and 5-FU, the mean average tumor volume was smaller than the group which treated only with 5-FU, and the difference was statistically significant (*P* < 0.05).

### The effects of 5-FU combined with Abnormal Savda Munziq on the expression of ALT, AST and TNF-α in serum

Comparing with the control group, the TNF-α expression in mice serum in the model group and 5-FU group were decreased significantly (*P* < 0.05); TNF-α expression in mice in the 5-FU + ASMq.H group and 5-FU + ASMq.M group were increased significantly (*P* < 0.05); the expression of ALT and AST in mice in the 5-FU group and different doses groups of 5-FU + ASMq were increased significantly (*P* < 0.05). Comparing with the model group, TNF-α expression in mice in the 5-FU group was decreased significantly (*P* < 0.05); TNF-α expression in mice in different doses groups of 5-FU + ASMq were increased significantly (*P* < 0.05); the content of ALT and AST in mice in the 5-FU group and different doses groups of 5-FU + ASMq were increased significantly (*P* < 0.05). Comparing with the 5-FU group, TNF-α expression in mice in different doses groups of 5-FU + ASMq were increased significantly (*P* < 0.05); the expression of ALT and AST in mice in different doses groups of 5-FU + ASMq were decreased significantly (*P* < 0.05). These results were summarized shown in Table 4.

**Table 4:** The effects of 5-FU combined with Abnormal Savda Munziq on the expression of ALT, AST and TNF-α in mice serum ($\overline{x}$±*s*)

Groups	TNF-α(pg/ml)	ALT(pg/ml)	AST(pg/ml)
Control	17.874±1.561	84.800±1.063	48.500±0.509
Model	15.837±0.981^ac^	80.400±4.014^c^	48.280±0.621^c^
5-FU	14.630±1.120^ab^	517.925±23.055^ab^	240.165±6.725^ab^
5-FU+ASMq.H	21.718±0.991^abc^	208.260±15.423^abc^	153.370±0.985^abc^
5-FU+ASMq.M	26.906±0.654^abc^	130.175±15.066^abc^	79.610±1.053^abc^
5-FU+ASMq.L	18.520±1.223^bc^	409.995±46.607^abc^	207.075±9.730^abc^

VS control group, ^a^ *P*<0.05;VS model group, ^b^ *P*<0.05 VS 5-FU group, ^c^ *P*<0.05

### The effects of 5-FU combined with Abnormal Savda Munziq on the protein expression of HPV16 E2 and TGF-β1 in mice bearing U27 cell-induced tumors

**Fig. 3:**
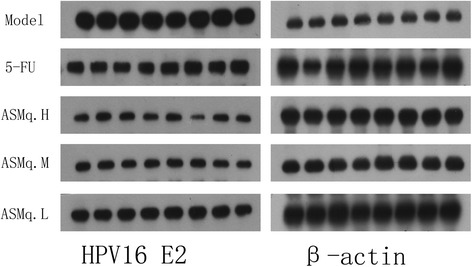
The effects of 5-FU combined with Abnormal Savda Munziq on the protein expression of HPV16 E2 in mice bearing U27 cell-induced tumors

**Fig. 4:**
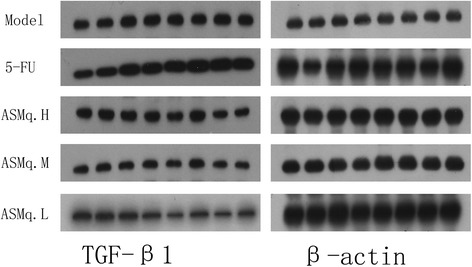
The effects of 5-FU combined with Abnormal Savda Munziq on the protein expression of TGF-β1 in mice bearing U27 cell-induced tumors

Compared with the model group, the level of protein expression of HPV16 E2 and TGF-β1 in tumors in the 5-FU group and different doses groups of 5-FU + ASMq were decreased significantly (*P* < 0.05). Compared with the 5-FU group, the protein level of HPV16 E2 and TGF-β1 in tumors in different doses groups of 5-FU + ASMq were also decreased significantly (*P* < 0.05). These results were shown in Fig. 3, Fig. 4 and Table 5, respectively.

**Table 5:** The effects of 5-FU combined with Abnormal Savda Munziq on the protein expression of HPV16 E2 and TGF-β1 in mice bearing U27 cell-induced tumors ($\overline{x}$±*s*)

Groups	HPV16 E2	TGF-β1
Model	1.829±0.115	1.270±0.232
5-FU	1.255±0.171^a^	1.058±0.100^a^
5-FU+ASMq.H	0.656±0.089^ab^	0.536±0.086^ab^
5-FU+ASMq.M	0.685±0.060^ab^	0.556±0.040^ab^
5-FU+ASMq.L	0.788±0.158^ab^	0.698±0.100^ab^

VS model group,^a^ *P*<0.05;VS 5-FU group,^b^ *P*<0.05

### Results of pathological analysis

**Fig. 5:**
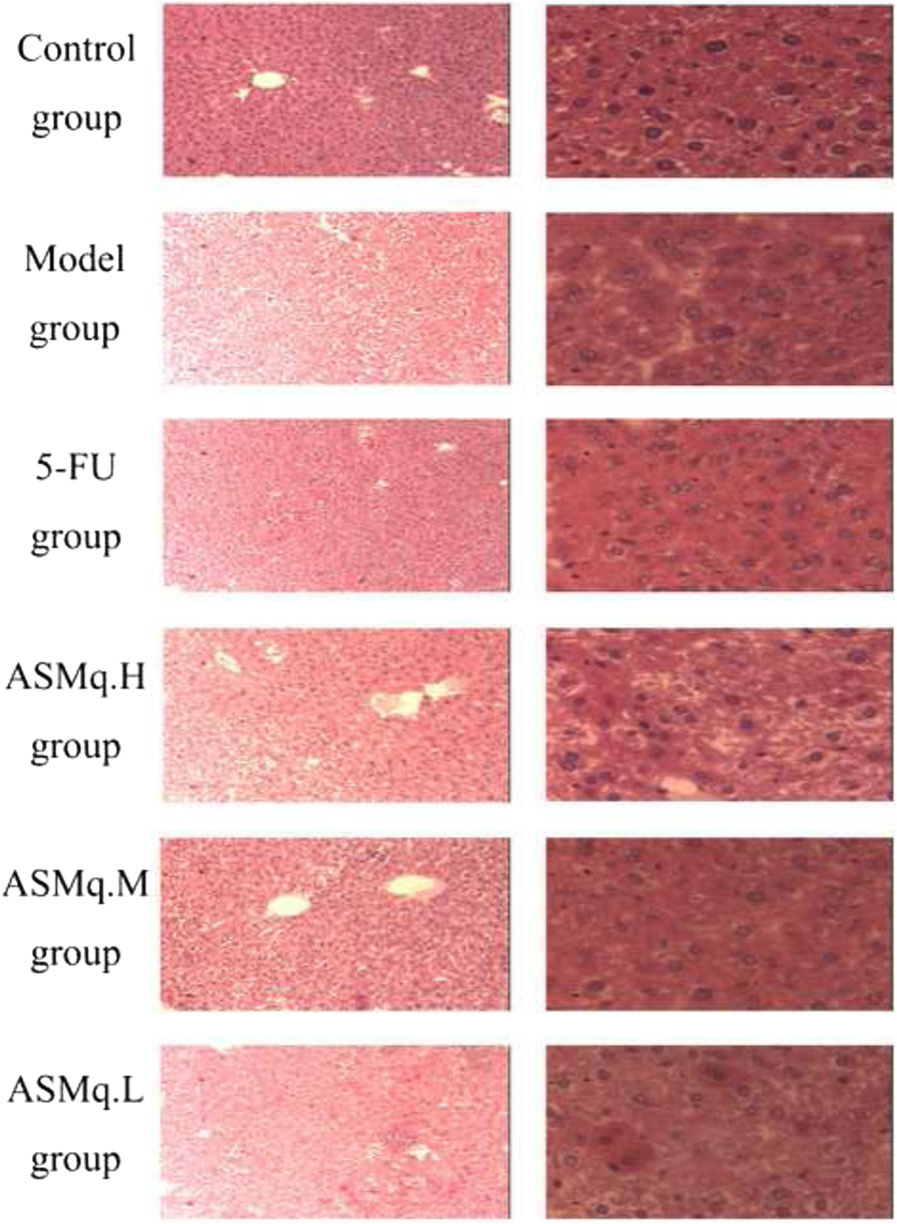
Liver pathology examination

The pathological changes in the liver from all experimental groups were analyzed by HE staining. From the histological sections in Fig. 5. In the control group, the liver tissue structure was normal, and no pathological changes were found under low power lens or high power lens. In the model group, the liver tissue structure was not distinguished under low power lens, and the liver cells generally swelling to be granular or eosinophilic change. According to high power lens, the liver structure was not distinguishable, and the liver cells generally swollen and changed into granules. In the 5-FU group, the liver tissue structure was not distinguishable, and the cells were generally swollen and changed into granules, or had diffuse eosinophilic change, and the central vein was dilated. Under high power lens, the structure of liver cells was not distinguishable, and the cells were generally swollen and changed into granules, or having diffuse eosinophilic change. In the 5-FU + ASMq.H group, under low power lens, the structure of liver cells was not distinguished, and the cells were generally swollen and changed into granules, or having diffuse eosinophilic change. Under the high power lens, the structure of liver cells was not distinguishable, and the cells were generally swollen and changed into granules. In the 5-FU + ASMq.M group, under low power lens, the liver tissue structure was normal, and the liver cells showed slightly swollen and changed into granules, or diffuse eosinophilic change. Under high power lens, swelling of the liver cells showed granular degeneration or scattered eosinophilic degeneration, and liver sinus were not distinguished. In the 5-FU + ASMq.L group, under the low power lens, the liver tissue structure was not distinguished, and the cells generally swelled and became granular or scattered eosinophilic change, and focal necrosis were found. Under the low power microscope, the liver structure is not clear, cell swelling or granular scattered eosinophilic degeneration, and liver sinus were not distinguishable.

## Discussion

Traditional Uyghur medicine shares the same origin of Greco-Arab medicine. According to traditional Uyghur medicine, nearly all diseases are caused by abnormal Hilits (syndromes), which result from imbalanced dynamic homeostasis of normal Hilits (humors): Kan, Phlegm, Safra, and Savda [11]. With this theory, abnormal Savda (syndrome) is predominant and is always the final condition of other abnormal Hilits. abnormal Savda is frequently associated with chronic and complex diseases, e.g. type II diabetes, cardiovascular disease, asthma, malignant tumors, and depression, particularly at advanced stages [12].Uyghur medicine is an important part of Chinese medicine, which provides unique approaches for the treatment of cancers and other complex diseases. A typical example is the Abnormal Savda Munziq [13], which is widely used as a cancer treatment or chemotherapy adjuvant in Uyghur clinical applications.

With consideration the compatibility of Chinese traditional medicine with other medicines, an innovative approach was used to combine western and Uyghur medicines to achieve the goal of reducing toxicity while maximizing efficacy. 5-FU is an anti-pyrimidine antimetabolite that is widely used in a variety of tumor chemotherapies. However, clinical use has revealed a major issue: the drug can cause strong immune inhibition and even liver damage. Therefore, reducing the side effects of chemotherapy and improving patients’ health conditions have become critical to the treatment of tumors. The results of this study suggested that the tumor inhibition rate of 5-FU combined with a high or medium dosage of Abnormal Savda Munziq is higher tha than which used alone, indicated that Abnormal Savda Munziq can enhance the anti-tumor effects of 5-FU.

TNF-α is a pleiotropic cytokine produced by the activation of macrophages and monocytes. It has a highly selective cell killing function, which can kill cancer cells while has no risk of damage to normal cells [14, 15]. TNF-α can directly kill tumor cells by activating T lymphocytes and stimulating the human body toproduce cytokines and antibodies. It also induces tumor cell apoptosis by acting on vascular endothelial cells [16]. TGF-β1 [17–19] is acytokine that is produced by many types of cells such as lymphocytes and monocytes, which is known to inhibit cytokine production. It can also inhibit the production of IFN-γ and TNF-α from peripheral blood mononuclear cells (PBMC). The results of this study suggested that TNF-α content in the serum was at a low level, while the protein expression of TGF-β1 in tumor tissue was at a high level in our tumor model group. With the combination of 5-FU and Abnormal Savda Munziq, the level of TNF-α is elevated in the serum, while the TGF-β1 expression is reduced. This suggested that Abnormal Savda Munziq may diminish the inhibiting effect of TGF-β1 on IFN-γ and TNF-α in PBMC, thereby increasing the concentration of TNF-α and enhancing anti-tumor effects [20, 21].

As the main regulatory protein of HPV DNA replication and gene expression, high-risk HPV E2 protein can repress cell mitosis and, therefore, cause metaphase-specific apoptosis. This process occurs regardless of oncogenes E6 and E7. Cells with high-risk HPV E2 protein expression often show polyploidy, abnormal chromosomal separation, centrosome amplification and increasing instability of the cell genome. HPV-encoded E2 protein may induce the integration of virus DNA and the human genome when combined with host HPV infection cytokines. This will effectively promote the integration of the virus and the host genome, which plays an important role in the oncogenic potential of high-risk HPV [22].

Immune function is closely related to the development of tumors, and cancer patients often experience immune dysfunction. This low immune function is more obvious in patients undergoing chemotherapy. The experimental results show that 5-FU damaged the body’s immune organs by suppressing tumors, and reducing the spleen and thymus indices. After applying different doses of Abnormal Savda Munziq in combinated with 5-FU, the spleen and thymus indices were significantly increased, which indicated that Abnormal Savda Munziq could reduce the damage of chemotherapy drugs to immune organs and protect the body’s immune system to a certain extent.

AST and ALT are mainly distributed in liver cells. ALT expression is a sensitive indicator of liver damage, when clinically meaningful increased enzyme activity is observed in the cytoplasm. AST, which occurs mostly in the mitochondria but can also sometimes also be found in the cytoplasm, can reflects the cardiac function index. An elevated ALT index indicates more liver cell damage and enhanced membrane permeability, while an elevated AST index suggests mitochondrial damage. The results showed that, after using 5-FU, AST and ALT levels in serum were significantly increasing, with combined treatment with Abnormal Savda Munziq, the two indexes were significantly decreasing. This suggested that Abnormal Savda Munziq may potentially protect the liver and heart during chemotherapy treatment. In addition, liver biopsy results showed that liver damage was significantly reducing after treatment combined with Abnormal Savda Munziq, which further suggested that Abnormal Savda Munziq had a protective effect on the liver.

## Conclusions

In summary, when the chemotherapy drug 5-FU was used combined with Abnormal Savda Munziq, its tumor inhibition efficacy was enhanced while the damage of the liver and immune organs was reduced. Therefore, treatment efficacy of 5-FU combined with abnormal savda munziq is increased and toxicity is reduced.

## Abbreviations

ASMq: Abnormal Savda Munziq
ASMq.H: Abnormal Savda Munziq high dose
ASMq.M: Abnormal Savda Munziq medium dose
ASMq.L: Abnormal Savda Munziq low dose
5-FU: Fluorouracil
